# Diversity analyses in two ornamental and large-genome *Ranunculaceae* species based on a low-cost Klenow NGS-based protocol

**DOI:** 10.3389/fpls.2023.1187205

**Published:** 2023-06-09

**Authors:** Matteo Martina, Alberto Acquadro, Ezio Portis, Lorenzo Barchi, Sergio Lanteri

**Affiliations:** Dipartimento di Scienze Agrarie, Forestali e Alimentari (DISAFA), Plant Genetics, University of Turin, Grugliasco, Italy

**Keywords:** K-seq, genotyping, ornamentals, *Ranunculus asiaticus*, *Anemone coronaria*, fingerprinting, NGS

## Abstract

Persian buttercup (*Ranunculus asiaticus* L.) and poppy anemone (*Anemone coronaria* L.) are ornamental, outcrossing, perennial species belonging to the *Ranunculaceae* family, characterized by large and highly repetitive genomes. We applied K-seq protocol in both species to generate high-throughput sequencing data and produce a large number of genetic polymorphisms. The technique entails the application of Klenow polymerase-based PCR using short primers designed by analyzing k-mer sets in the genome sequence. To date the genome sequence of both species has not been released, thus we designed primer sets based on the reference the genome sequence of the related species *Aquilegia oxysepala* var. *kansuensis* (Brühl). A whole of 11,542 SNPs were selected for assessing genetic diversity of eighteen commercial varieties of *R. asiaticus*, while 1,752 SNPs for assessing genetic diversity in six cultivars of *A. coronaria*. UPGMA dendrograms were constructed and in *R. asiaticus* integrated in with PCA analysis. This study reports the first molecular fingerprinting within Persian buttercup, while the results obtained in poppy anemone were compared with a previously published SSR-based fingerprinting, proving K-seq to be an efficient protocol for the genotyping of complex genetic backgrounds.

## 1 Introduction

The Ranunculaceae family is classified as a basal eudicotyledonous group and includes approximately 2500 species distributed across 53 genera (Jiang et al., 2017). Over the centuries, this botanical family has been the subject of systematic investigations due to its intriguing position within flowering plants and its notable variation in vegetative and reproductive structures. Breeding efforts have been mainly focused on the exploitation of the ornamental potential of two species: *Anemone coronaria* L., commonly known as poppy anemone (2n=2x=16; estimated genome size: 9.08Gb; Martina et al., 2022a) and Ranunculus asiaticus L., commonly known as Persian buttercup (2n=2x=16; estimated genome size: 7.6Gb; Martina et al., 2022b). Both species are outcrossing, perennial crops marketed as cut flowers and potted or garden plants. In poppy anemone modern cultivars have been primarily selected for the production of cut flowers, and breeding efforts has been focusing on product uniformity, obtainment of new flower colors, adaptation to long photoperiods, precocity, and longevity of vase life. Cut flower varieties must bloom early, possess sturdy stems and large multi-sepaled flowers, while garden and potted plant types must exhibit compact growth habits, upright leaves with short petioles and abundant and coordinated flowering. In Persian buttercup, selection for cut flowers has been mainly focusing on flower diameter, morphology, and color. The Pon-Pon^®^ varieties, created by the Company ‘Biancheri Creations’ through a long breeding and micropropagation process, have become increasingly important in the market due to their unique bicolor and jagged petal shape. Other innovative Ranunculus varieties, such as those with a “green center” are also gaining popularity. Furthermore, in both species a key breeding objective has been also the resistance/tolerance to biotic and abiotic stresses, and since the commercial varieties are vegetatively propagated through rhizomes, the easy handling of roots at harvest and rhizome storability are highly desirable for commercial profitable production. In this context the availability of molecular markers and the application of novel genomics tools may play an essential role to accelerate breeding programs.

The advent of high-throughput next-generation sequencing (NGS) methods has resulted in a shift from fragment-based polymorphism identification to sequence-based single nucleotide polymorphism (SNP) detection, thereby augmenting the number of markers available for population genetics research and expediting the development of high-density genetic maps and the discovery of genes and quantitative trait loci (QTLs) (Sahu et al., 2020). The sustained reduction in sequencing costs has facilitated the formation of genomic consortia for the release of reference genomes in a diverse range of plant species (Jaillon et al., 2007; Sato et al., 2012; Hibrand Saint-Oyant et al., 2018; Raymond et al., 2018; Acquadro et al., 2020; Barchi et al., 2021; Nakano et al., 2021; Nashima et al., 2021), making it possible to design genotyping assays that offer potent tools for next-generation plant breeding (Sim et al., 2012; Víquez-Zamora et al., 2013; Barabaschi et al., 2016; Vukosavljev et al., 2016; van Geest et al., 2017; Barchi et al., 2019). Nevertheless, the efficacy of these assays is dependent on the availability of prior genomic information and their application to “orphan” species possessing large, heterozygous, and highly repetitive genomes may prove challenging, and even high-depth sequencing may not guarantee the quality of assembly for such species (Unamba et al., 2015). An alternative approach is represented by reduced-representation sequencing (RRS) techniques, which can generate genome-wide high-throughput sequencing data and obtain a significant number of genetic polymorphisms, representative of the entire genome information of the species (Wright et al., 2019). RRS based on Restriction enzymes-based techniques are generally applicable to any genetic background (Davey and Blaxter, 2010; Barchi et al., 2012; Peterson et al., 2012; Acquadro et al., 2017; Jordon-Thaden et al., 2020; Sunde et al., 2020; Toppino et al., 2020), mainly requiring the selection of the appropriate enzyme, making them a common approach in non-model species and small to medium-scale projects. However, such techniques but require a license which increasing the genotyping costs (Barchi et al., 2019). To overcome these limitations, non-restriction-based RRS methodologies have been developed, such as rAmpSeq (Buckler et al., 2016), which is based on the amplification and sequencing of repetitive regions, have been developed. Building on this concept, the K-seq protocol was developed by Mata-Nicolás et al., 2021 and Ziarsolo et al., 2021, which relies on the amplification of genomic regions using two steps of Klenow amplification with short oligonucleotides, followed by standard PCR and Illumina sequencing. The design of primers in this protocol can also be performed using the genome sequence of a closely related species by analyzing k-mer sets, making it applicable to genome-orphan species as well. Furthermore, the license-free nature of K-seq and low library preparation costs make it a valuable alternative to GBS and ddRADseq.

To date, the reference genomes of both Poppy anemone and Persian buttercup have not been released and the application of DNA-based techniques has been limited (Nissim et al., 2004; Fang and Paz, 2005; Laura et al., 2006; Martina et al., 2022a; Martina et al., 2022b). Here, we applied the K-seq protocol on these two species using primer sets obtained from the high-quality reference genome of the closely-related *Aquilegia oxysepala* var. *kansuensis* (Brühl.; (Xie et al., 2020)). The protocol was applied to breeding material commercialized by Biancheri Creazioni, specifically eighteen cultivars of *R. asiaticus* and six cultivars of *A. coronaria*, the latter being validated with previously published Single Sequence Repeats (SSR)-based fingerprinting (Martina et al., 2022a). Furthermore, the fine-tuning of the protocol showed to be cost-effecting for routine genotyping application.

## 2 Materials and methods

### 2.1 Plant material and DNA extraction

Eighteen market varieties of *R. asiaticus*, known for their production of double and Pon-Pon^®^ flowers, and six diploid cultivars of *A. coronaria*, which have been previously characterized using SSR markers (Martina et al., 2022a), were procured from “Biancheri Creazioni” as representative samples of their commercial breeding stock (as indicated in **Supplemental Table 1**). Genomic DNA was extracted from frozen leaves with the Plant DNA Kit (E.Z.N.A.^®^), following manufacturer’s instructions. DNA quality was assessed through the NanoDrop™ 2000 spectrophotometer (ratio 260/280 between 1.8 and 2.0; ratio 260/230 > 2.0), and the Qubit^®^ 2.0 Fluorometer was used for DNA quantification.

### 2.2 Primers’ design and library preparation

The recently published chromosome-scale reference genome of *Aquilegia oxysepala* var. *kansuensis*, a closely-related species to both species in study and belonging to the same family, was retrieved from the NCBI database. The Primer Explorer 2 pipeline (https://github.com/bioinfcomav/primer_explorer2) and used for oligonucleotide sets identification. Briefly, the 1000 most abundant k-mers with a GC content between 35 and 75% were selected and combined to create sequence sets (8-9bp each) suitable for PCR amplification. The pipeline provided a virtual PCR, producing a report (**Supplemental Table 2**) which included a number of predicted single and repetitive products for each primer pair within each oligonucleotide set. Three primer pairs were selected for *A. coronaria* and *R. asiaticus* genotyping, according to the predicted number of amplified fragments in *A. oxysepala* var. *kansuensis* (**Supplemental Table 3**). Library preparation was performed according to Mata-Nicolás et al., 2021 and Ziarsolo et al., 2021, halving enzymes concentrations and doubling reaction times in order to reduce genotyping costs. Briefly, the genomic DNA was subjected to denaturation and annealing at 37°C using forward primers. Then, the large Klenow fragment of DNA polymerase I (New England Biolabs, MA, USA) was utilized for the initial round of DNA synthesis. Following inactivation of the polymerase at 75°C, the remaining single-stranded DNA was degraded at 37°C using Exonuclease I (New England Biolabs, MA, USA) and inactivated at 80°C. The above steps were repeated with the reverse primers. The reaction product was employed as a template in a standard 15-cycle enrichment PCR utilizing in-house Illumina Nextera primers, which featured dual indexes. Finally, the indexed samples were combined and the libraries were size-selected (300-700 bp) through gel excision and extraction (E.Z.N.A.^®^ Gel Extraction Kit), as per the manufacturer’s protocol.

### 2.3 Sequencing data analysis and SNPcalling

Libraries were Illumina sequenced (150PE) on NovaSeq 6000 (Illumina, San Diego, CA). After demultiplexing, reads were trimmed and cleaned using fastp (Danecek et al., 2021) and mock references (*catalogs*) were produced through the GBS-SNP-CROP pipeline (Melo et al., 2016). The six genotypes in study were used as input for *A. coronaria*, while for *R. asiaticus* just two genotypes, showing the highest sequencing output, and representative of the cultivars characterized by the so called Pon-Pon^®^ (Pon-Pon^®^ - 929) and double flower (Success^®^ - 311), were used. Sequences for each genotype were mapped to the catalog file using the Burrows-Wheeler Aligner (BWA, v0.7.17) program and the ‘mem’ command with the default parameters (Li and Durbin, 2009). SNP calling was performed using Bcftools mpileup (v1.17; Danecek et al., 2021) and SNPs were filtered to remove those with a mean depth below 20 (MEAN(FMT/DP)>=20), a mean QUAL higher than 20 (MEAN(FMR/GQ)>20), no more than 20% of missing data (F_MISSING=<0.2), and a MAF >= 0.05. Markers were named according to the catalog sequence in which they were identified. Sequencing reads were aligned also on the reference genome of *A. oxysepala*, following the same protocol mentioned above. After reads aligning, a window size of 100kb was set in the Mosdepth script (Pedersen and Quinlan, 2018). Putative k-mer fragments were retrieved through the K-seq bioinformatic pipeline for *Aquiligea*’s genome, and a 100kb was applied using *bedtools windows* and *bedtools coverage* (v2.31.0; Quinlan and Hall, 2010), to calculate the bin coverage across the genome.

### 2.4 Molecular fingerprinting and data analysis

After quality filtering, the identified markers were imported into MEGA11 software (Tamura et al., 2021) and used to construct a UPGMA-based dendrogram (Sneath and Sokal, 1973) with 1,000 bootstraps for *A. coronaria*, consistently with the previously reported SSR-based philogeny, and a NJ-based dendrogram for *R. asiaticus*. Principal component analysis (PCA) was also performed (SNPRelate v1.32.1; Zheng et al., 2012), displaying the multi-dimensional relationship between genotypes, and the two axes were graphically plotted, according to the extracted eigenvectors. To compare the previously published SSR-based fingerprint with the novel one, Mantel test (Mantel, 1967) was performed to evaluate the correlation index (r), between the two similarity matrices.

## 3 Results and discussion

### 3.1 Primers’ development and sequencing data analysis

The versatility of K-seq as a technique has been well established, as demonstrated by its ability to provide reliable genotyping results in closely related species using primer sets designed for a specific species (Mata-Nicolás et al., 2021; Ziarsolo et al., 2021). This makes K-seq particularly useful when working with genome-orphan species.

Sampling genomic loci - To date, the *Anemone* and *Ranunculus* genera lack high-quality reference genomes. As a result, primer mining was performed using the Primer Explorer 2 pipeline, which was based on the recently released genome sequence of *A. oxysepala* var. *kansuensis*, the most closely related species. The pipeline utilized the analysis of the 1000 most abundant k-mers, enabling the selection of three sets of primer pairs that provided the greatest number of amplicons in *A. oxysepala* (**Supplemental Table 3**).

Sequencing throughput - Overall, ~26M reads for the six cultivars of *A. coronaria* and ~164M reads for the 18 *R. asiaticus* cultivars were produced, generating a total of 29.3Gb of raw data. The number of reads obtained per sample showed variability among accessions. In *Anemone*, reads output ranged between 1.2 and 6.8 million, while in *Ranunculus* between 1.1 and 18.8 million (**Supplemental Table 4**). The presence of heterogeneity in the number of reads per sample has been previously reported by Mata-Nicolás et al., 2021 and Ziarsolo et al., 2021. This phenomenon is believed to be the result of the absence of DNA quantification of PCR-amplified products prior to library pooling. In order to mitigate this issue and maintain the cost-effectiveness of the protocol, we conducted a direct quantification of the library using electrophoresis and densitometric analysis of band intensity, as well as comparison to a standard curve generated from DNA of known concentrations (ranging from 20 to 100 ng). Despite these efforts, our results indicated that this approach was not sufficient for standardizing the number of reads in the K-seq sample pooling step. Indeed, this step is crucial for producing standardized data and may require additional purification techniques such as SPRI bead purification after library enrichment to remove primer dimers and facilitate accurate quantification of products using fluorescence-based methods. According to our evaluations and in line with the current consumables pricing in our lab, by halving enzymatic concentrations and reducing the enrichment reaction volume to 20ul (the original protocol suggests 50ul), the *per sample* cost of the protocol can be decreased by a broad 50%, passing from 12€ to 6€ per library. In this scenario, we believe that SPRI beads might be taken into account for routine genotyping application of k-seq protocol, even with large sample sets. Such optimization allows for library quantification prior to samples pooling, requiring extra costs represented by fluorometer quantification. However, it must be noted that, after our adjustments, SPRI beads introduction in the protocol might impact on the single sample cost for a broad 30%, leaving space for their evaluation according to project budget and overall experimental design.

Not only library preparation, but also sequencing costs impact on project budget. In our experiment the technique was found to be reliable without SPRI optimizations and, with an average of 2M reads for *A. coronaria* and 4M reads for *R. asiaticus*, our results suggest that a sequencing output of approximately 1Gb per sample represents a cost-effective solution for high-quality genotyping of complex species. With the current sequencing costs, such coverage appears to be reasonable for large-genome species, but might be considerably lowered for smaller ones.

### 3.2 SNP mining

**Figure 1:**
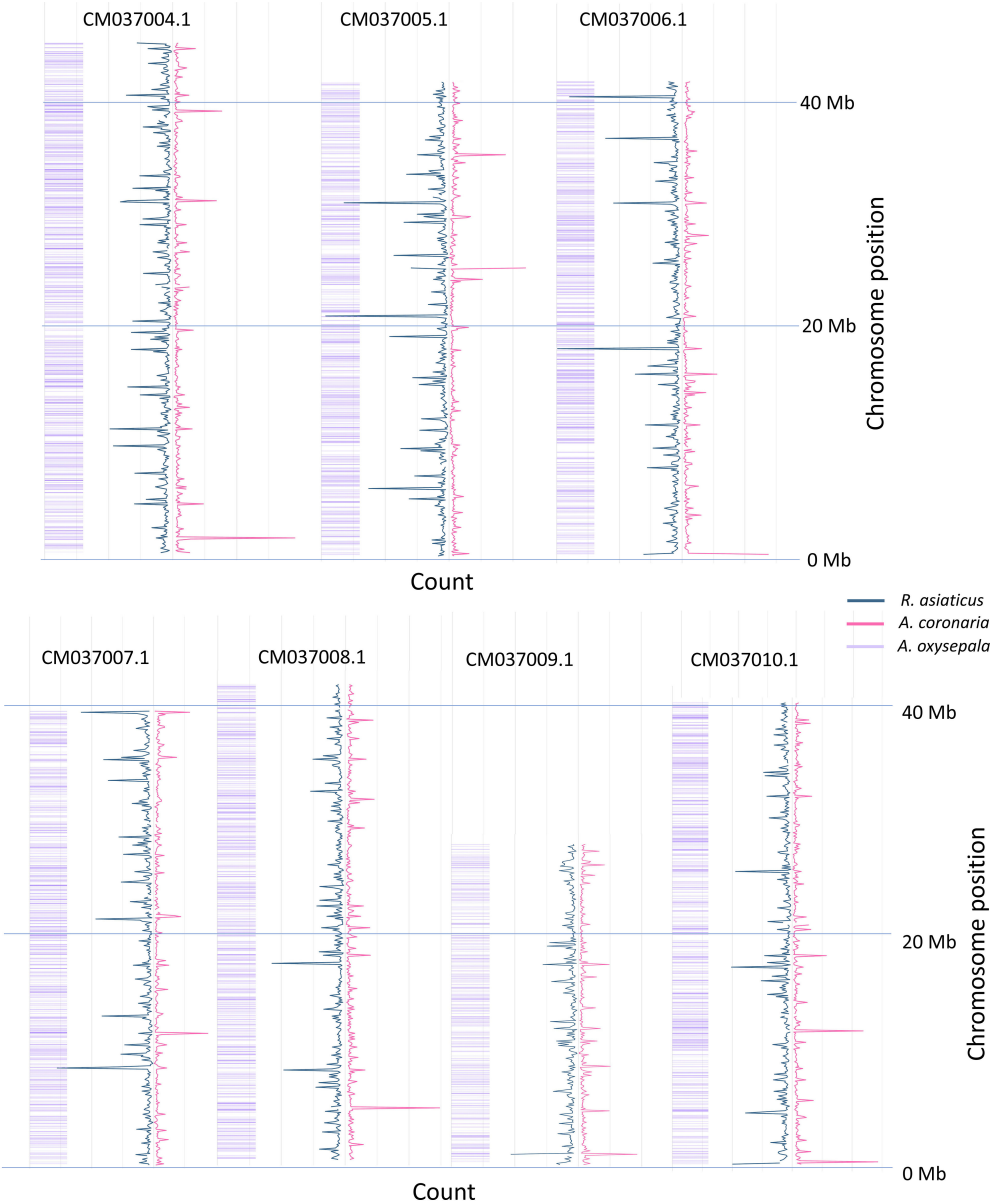
Genome-Wide coverage of the sequenced reads on the A. oxysepala genome. Only the seven chromosomes are presented for conciseness; *R. asiaticus* in blue, while *A. coronaria* in pink. The values for A. coronaria, of which only 6 samples were sequences, were re-scaled of a 3x factor, allowing a clear comparison in the figure. The predicted K-seq fragments originated by the three sets of primers in *A. oxysepala* are reported in purple.

Mock references (*catalogs*) were produced for the two species through the GBS-SNP-CROP *pipeline*. A whole of 2,374,786 contigs catalog was assembled in *A.* while the *R. asiaticus* the catalog included 2,416,619 contigs. Cleaned reads were back-aligned on the mock references and 629,004 and 8,021,225 SNPs were called, subsequently reduced to 1,752 and 11,542 after filtering for *A. coronaria* and *R. asiaticus* respectively, and were then used for genetic diversity analyses. Heterozygosity was calculated (**Supplemental Table 4**), identifying a clear difference between the two species. The average heterozygosity was observed to be 0.5 in *Anemone* cultivars and was estimated at 0.34 in *Ranunculus*. These differences may be attributed to the distinct breeding practices applied for cultivar selection in the two species. Specifically, poppy anemone cultivars are usually produced by cross-pollinating selected parental lines, resulting in a substantial degree of morphological heterogeneity and without a focus on reducing heterozygosity. Conversely, Persian buttercup cultivars are commonly propagated by cloning and selected for their superior market appeal from a diverse range of segregating crosses. In this species, breeding strategies have also been directed towards reducing heterozygosity with the objective of achieving stable crosses (personal communication with Biancheri Creazioni). Although both species are known to possess a highly heterozygous genetic background (Fang and Paz, 2005; Laura et al., 2006; Martina et al., 2022a; Martina et al., 2022b), the lower heterozygosity observed in Persian buttercup may be attributed to the breeding selection implemented at Biancheri Creazioni. The expected distribution of the k-mer fragments on the *A. oxysepala* genome was obtained through the K-seq native pipeline, predicting the three selected primer sets to produce a good coverage of all the seven chromosome of the species (**Figure 1**). Subsequently, the raw reads were aligned to the reference genome of *A. oxysepala*, and their distribution was compared to the predicted k-mer fragments to assess the amplified fragments in the two ornamental species (**Figure 1**). Approximately, 20% of the reads mapped to *Aquilegia*’s genome in both species, while an average of 90% of the reads were successfully aligned to the two catalogs. As shown in **Figure 1**, the reads that mapped to the reference genome exhibited a broad coverage across the entire genome, consistent with the predicted fragments. Differences in overall coverage were observed, likely influenced by the number of individuals in the two panels and discrepancies between the two sequencing outputs. Nevertheless, it is plausible that the mapping reads may represent essential shared information among the three Ranunculaceae species, which diverged approximately 60 million years ago (Zhai et al., 2019). Genetic variation in cultivars of poppy anemone.

**Figure 2:**
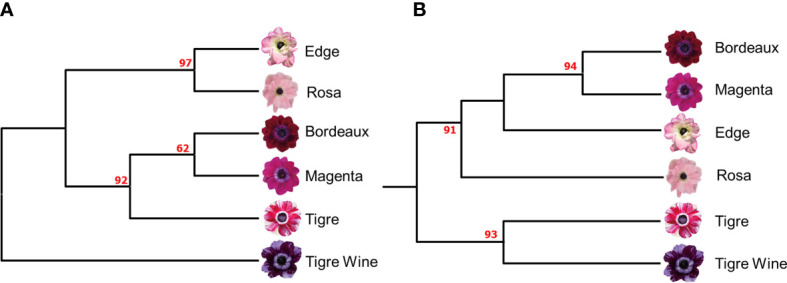
**(A)** UPGMA dendrogram based on 1,752 SNPs identified by the K-seq protocol **(B)** UPGMA dendrogram based on 62 SSR (adapted by Martina et al., 2022b). Bootstrap values (%) are reported in red.

An UPGMA dendrogram of the six *A. coronaria* cultivars was produced (**Figure 1A**), and the detected genetic relationships among the cultivars were in good agreement with the previously published SSR-based fingerprint (**Figure 1B**; Martina et al., 2022a), since Mantel correlation test highlighted a good fit between the similarity matrices (0.85). Two major clades were identified following the application of the K-seq protocol (**Figure 2A**). In the first branch, the cultivars “Rosa”, and “Edge” clustered with a bootstrap probability of 97%. In the second branch, the cultivars “Bordeaux”, “Magenta” and “Tigre” clustered with a bootstrap value of 92%. The cultivar “Tigre wine” was identified as the most distant variety, in contrast with the previously published SSR based finger-print that grouped it with “Tigre” (**Figure 2B**). As previously reported (Xie et al., 2020), this demonstrates the effects associated with different genotyping platforms on the data interpretation. However, the two topologies were overall highly related, suggesting that K-seq represents a valuable tool to generate genotypic profiles. Furthermore, the K-seq procedure represents a valid and alternative approach to the more costly and time-consuming SSR-assays development and to the patented GBS-like approaches relying on restriction enzymes.

### 3.3 Genetic variation in cultivars of Persian buttercup

**Figure 3:**
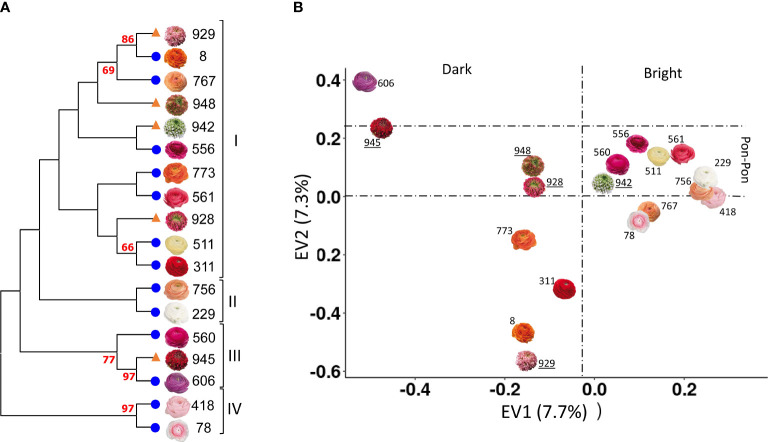
NJ dendrogram **(A)** and PCA analysis **(B)** of the eighteen varieties of *R. asiaticus*, based on 11,542 SNPs. Bootstrap values (%) higher than 60 are reported in red. Pon-Pon varieties are represented with an orange triangle in the NJ dendrogram **(A)**, and their names are underlined in the PCA **(B)**; on the other hand, double-flowered varieties are represented with a blue circle in the NJ dendrogram **(B)**, in order to make it possible to easily discriminate between the two groups.

The same sets of primer used in poppy anemone was applied to obtain a NJ dendrogram of the eighteen cultivars of *R. asiaticus* (**Figure 3A**). Since no prior phylogenetic studies have been reported for Persian buttercup, our results are the first ones obtained in this species. A PCA analysis was performed on the genotyping data, highlighting that the first two axes (**Figure 3B**) explained 7.7 and 7.3% of the overall genetic variation. In **Figure 3B** the cultivars pairs “606”/”945”, and “929”/”8” clustered according to the first PCA component and were clearly differentiated from the others, while the cultivars “418” and “78” clustered according to the second one. Furthermore, with the exception of the cultivar “945”, the NJ-based phylogeny clustered the cultivars defined Pon-Pon^®^ in Clade I (**Figure 3A**), and the first component of the PCA grouped the varieties with brighter flower colors in the right side of the scatter plot and the darker ones in the left side.

## 4 Conclusions

The application of K-seq in two *Ranunculaceae* species, characterized by large and highly repetitive genomes, has here been reported for the first time. The species, whose sequence has yet to be released, were found to be suitable for this genotyping protocol, which can be easily applied even in “orphan” species by utilizing genomic information from closely-related species. In Zhai et al., 2019, investigated the phylogeny and evolution of the family by sequencing the chloroplast genome of 35 species, representing 31 genera of the 14 tribes of the Ranunculaceae family. With the exception of Glaucideae, Hydrastideae, and Coptideae, which diverged earlier (89.9 Mya), molecular clock analysis highlighted how the first divergence within the Ranunculaceae core occurred around 66.3 Mya, followed by subsequent independent branch-splitting event in the two lineages. As *Ranunculus* sp., *Anemone* sp., and *Aquilegia* sp. belong to the Ranunculaceae core, the applied methodology might be robust enough for comparing species across different genus. Furthermore, by reducing the enzymatic concentration in the reaction, the cost of the library preparation was significantly reduced. As previously reported (Ziarsolo et al., 2021), the number of K-seq fragments generated by Klenow amplification can deviate from the expected amount due to mismatched primers. By assessing the number of generated fragments through agarose gel visualization, or by using microfluidic-based electrophoresis, the best primer set can be identified, and the generated data also allow for the selection of the best number of primers sets applied for genotyping. However, our experiment highlighted that the protocol was reliable without such optimizations, demonstrating that an expected sequencing output of approximately 1Gb per sample might be a cost-effective solution for achieving high-quality genotyping results in complex species. Our results confirm that K-seq is comparable or in some cases cheaper than GBS in terms of cost. In *A. coronaria* the estimated genetic diversity and the clustering of the eight cultivars in study were found consistent with those previously published and based on a SSR genotyping. Side by side this study provides the first assessment of genetic diversity in marketed varieties of *R. asiaticus* and novel information on their genetic relationships. The Pon-Pon^®^ varieties did not cluster separately from the others, and thus they do not appear genetically differentiated. As confirmed by the breeders of the Company ‘Biancheri Creations’, the peculiar flower trait not always showed to be stable in different environmental conditions and it is thus presumably attributable to epigenetic mechanisms, at least partly influenced by growth conditions.

The user-friendly nature of this technique, together with its cost-effectiveness, may find applications in cultivar fingerprinting and legal disputes, having the potential to facilitate the implementation of molecular-assisted breeding programs in this two complex species, promoting the efficient development of new cultivars and enabling the identification of the genetic factors underlying complex traits.

## Data availability statement

The datasets presented in this study can be found in online repositories. The names of the repository/repositories and accession number(s) can be found below: NCBI, PRJNA954204.

## Author contributions

Conceptualization, MM, LB, EP, and SL; methodology, MM, LB, and AA; software, MM, LB, and AA; validation, MM, LB, and EP; formal analysis, MM and EP; investigation, MM and EP; data curation, MM; writing—original draft preparation, MM and AA; writing-review and editing, LB, EP, and SL; visualization, AA and EP; supervision, EP and SL. All authors contributed to the article and approved the submitted version.
